# Examining preconditions for integrated care: a comparative social network analysis of the structure and dynamics of strong relations in child service networks

**DOI:** 10.1186/s12913-023-10128-z

**Published:** 2023-10-24

**Authors:** Mariëlle Blanken, Jolanda Mathijssen, Chijs van Nieuwenhuizen, Jörg Raab, Hans van Oers

**Affiliations:** 1 TRANZO - Scientific center for care and wellbeing, Tilburg University, 5000 LE Tilburg, PO BOX 90153, the Netherlands; 2 Department of Organization Studies, School of Social and Behavioral Sciences, Tilburg University, NL-5000 LE Tilburg, P.O. Box 90153, The Netherlands

**Keywords:** Strong relations, Stability, Critical relations, Gatekeepers, Key position, Child service delivery networks, Longitudinal multiple case studies, Social network analysis, QAP correlation procedure, Preconditions integrated care.

## Abstract

**Background:**

To help ensure that children and families get the right support and services at the right time, strong and stable relationships between various child service organizations are vital. Moreover, strong and stable relationships and a key network position for gatekeepers are important preconditions for interprofessional collaboration, the timely and appropriate referral of clients, and improved health outcomes. Gatekeepers are organizations that have specific legal authorizations regarding client referral. However, it is largely unclear how strong relations in child service networks are structured, whether the gatekeepers have strong and stable relationships, and what the critical relations in the overall structure are. The aim of this study is to explore these preconditions for integrated care by examining the internal structure and dynamics of strong relations.

**Methods:**

A comparative case study approach and social network analysis of three inter-organizational networks consisting of 65 to 135 organizations within the Dutch child service system. Multiple network measures (number of active organizations, isolates, relations, average degree centrality, Lambda sets) were used to examine the strong relation structure and dynamics of the networks. Ucinet was used to analyze the data, with use of the statistical test: Quadratic Assignment Procedure. Visone was used to visualize the graphs of the networks.

**Results:**

This study shows that more than 80% of the organizations in the networks have strong relations. A striking finding is the extremely high number of strong relations that gatekeepers need to maintain. Moreover, the results show that the most important gatekeepers have key positions, and their strong relations are relatively stable. By contrast, considering the whole network, we also found a considerable measure of instability in strong relationships, which means that child service networks must cope with major internal dynamics.

**Conclusions:**

Our study addressed crucial preconditions for integrated care. The extremely high number of strong relations that particularly gatekeepers need to build and maintain, in combination with the considerable instability of strong relations considering the whole network, is a serious point of concern that need to be managed, in order to enable child service networks to improve internal coordination and integration of service delivery.

## Highlights

More than 80% of the organizations in the network has strong relations.

Gatekeepers need to maintain an extremely high number of strong relations.

Child service networks need to cope with major internal dynamics.

These insights offer leads for optimizing how a network functions.

## Background

Integrated care is widely recognized as an approach to promote the ‘Triple Aim’ goals in health system reform: higher cost efficiency, improved quality of care, and improved health outcomes [1, 2]. It requires a holistic and an inclusive approach, seeking to build trusted relationships between organizations in the health system and respecting each organization as an equal partner [3, 4]. To achieve this, many countries have shifted key responsibilities for child welfare and healthcare service delivery (hereinafter referred to as child service networks) from the central to local levels of government [5–12]. These state reforms were meant to facilitate integrated care in families’ own environment by decompartmentalizing budgets and strengthening the relations between various child service organizations [11–16].

Strong and stable relationships facilitate trust as well as familiarity and enable fine-grained information exchange regarding clients’ conditions and effective treatment. This makes such relationships crucial for interprofessional collaboration, the timely and appropriate referral of clients, and improved health outcomes [12, 15–17]. They are vital to help ensure that children and families get the support and services they need from professionals with the required skills in an efficient manner [15, 18–22]. Relationships become stronger when organizations interact more frequently with each other, when the contact requires reciprocity in the exchange of resources, and when organizations are connected in more than one way due to multiple resources exchange relationships with each other [23–25]. Stability occurs as relations mature over time [26–28].

In practice, however, maintaining a high number of relations, especially strong relations, can be challenging for organizations [12]. Organizations have limited resources, energy, time and cognitive capacity and can therefore not maintain a large number of strong relations [29]. Maintaining many relations therefore carries the risk of inefficient and ineffective functioning of these child service networks. Nonetheless, child service networks generally consist of many organizations working across several sectors, such as mental healthcare, education, childcare and nursery, specialized youth care and community services [2, 12, 20, 21, 30–36]. Moreover, as networks are dynamic systems, it is to be expected that strong relations are continuously evolving and ending, networks therefore need to deal with internal dynamics [26, 29, 37]. Since the loss of relations leads to a loss of social capital, to an increase of fragmentation in care and ultimately affects service sustainability, integrated care cannot be guaranteed in networks with too few strong and stable relationships [16, 38, 39]. Consequently, there is a considerable risk that children and families in need do not get the right service at the right time or may even be overlooked and left untreated [40]. In effect, maintaining a crucial number of strong and stable relationships is a key challenge for the networks to be able to operate effectively.

Besides the presence of strong and stable relationships, how these relations are structured is critical for an effective functioning of the network as a whole [21, 25, 41–43]. This applies in particular to core organizations. Important core organizations in child service networks are organizations with a gatekeeper function. These gatekeeper organizations have specific legal authorizations regarding client referral, one of the core processes to ensure that the support services that children and families need are provided [19, 44–46]. Moreover, to optimize client referral and information flow between all organizations in the network, it is crucial to recognize the critical relations in the overall network structure. Critical relations are those relations in the network that form a bridge between (groups of) organizations within the network that otherwise would not be connected. In other words, any disruption to this bridge would result in a grave disruption to the flow of clients and information [29, 47]. Organizations that form these critical relations have a key position in the network.

Due to the scarcity of longitudinal comparative whole network research in the field of child service networks, it is largely unclear how strong relations in child service networks are structured, whether the gatekeepers have strong and stable relationships, and what the critical relations in the overall structure are [21, 30, 40–43, 48–56]. To further open the black box of integrated care and to enable child service networks to improve internal coordination and integration of service delivery, it is very important to understand the internal structure and dynamics of strong relations in a network [19, 30, 57–61]. Therefore, this study aims to identify the overall structure of strong relationships between organizations in child service networks, the critical relations in these networks, and the extent of relation stability over time, by using social network analysis. In particular, the study focuses on the relationships of organizations with a gatekeeper function.

## Methods

Since part of the collected data was used in previous publications of this study by the same authors, some elements of the methods have been described before [36, 37, 62].

### Research setting

The research field of this study is the societal and administrative context of the Dutch child welfare and healthcare service delivery system [36, p. 81, 37, p. 3]. Like many other countries, the Netherlands implemented welfare and healthcare state reforms that shifted key responsibilities from the central to local levels of government [5–10]. Since 2015, municipalities have become fully responsible for the child welfare and healthcare service delivery system [37, p. 3]. The gatekeepers are the centers for youth and family, general practitioners and child health care organizations, which means that they are legally authorized to commission child and youth services covered by the Child and Youth Act [46].

A comparative case study was conducted of three inter-organizational networks of child services in different-sized municipalities in the Netherlands [36, 62–64]. Network I was located in a midsize municipality (around 180,000 citizens), Network II in a small municipality (around 66,000 citizens), and Network III covered four very small municipalities that collaborate in providing child services (with 13,000–20,000 citizens per municipality, i.e., a total of about 60,000 citizens) [36, p. 81, 37, p. 3, 62, pp. 30–31].

### Data collection

The data of the three networks were collected at two points in time. The first data collection took place in the period of November 2017 to September 2018, the second in the period of April to September 2019. Both data collections consisted of two steps. First, semi-structured interviews with the network managers were conducted. The aim of the interviews was to determine the goals of the network, to define the boundaries of the network by determining the network members, and to select representatives of the network members as potential respondents for the online questionnaire. Second, an online questionnaire was fielded among the representatives of the network members [36, pp. 82–83, 37, p. 4, 62, p. 31].

### Research population and boundary specification

The study combined a nominalist and realist approach to network boundary specification [62, p. 31]. We defined a criterion to include organizations first (nominal approach) and then used the judgment of participating individuals in the network to determine the boundaries (realist approach) [62, 65]. The following definition of a network was used: *the network of child services consists of organizations that, according to the network manager, work with the local government to achieve the main network goal of the Child and Youth Act* [36, p. 81, 37, p. 3, 62, p. 31]. The research population consisted of organizations that participate in the child service networks, i.e., network members, with the representatives of the network members as the units of observation [66]. The respondents were employees who act as boundary spanners between organizations in the network [67, 68]. The network managers - the responsible managers of the municipalities’ child and youth support departments - were asked to identify the network members and to select the boundary spanners for each network [36, pp. 81–82, 37, p. 3, 62, p. 31]. The selection of network members, including boundary spanners, was verified by colleagues of the municipalities’ child and youth support department and compared to information on network members kept by the department’s administrative system [36, p. 82, 62, p. 31].

Since the individual professionals of some network members operated within a limited working area – such as school care coordinators, school attendance officers, general practitioners (family doctors) and organizations for childcare and nursery - we invited more than one boundary spanner from these network members. For example, in Network I there were a total of thirty general practitioners in the municipality. As the working area of one general practitioner was limited to a small part of the municipality, we invited them all to participate [36, p. 82, 37, p. 4, 62, p. 31].

For Network I, we also used a threshold for the selection of network members from the sector “specialized youth care organizations”. As a relatively large number of these organizations only had a few juveniles in treatment in one year and therefore held peripheral positions in the network, we selected only the organizations that had a minimum of six juveniles receiving care in 2017 (94 of 162 organizations) and in 2018 (92 of 172 organizations). This threshold is generally used for privacy reasons. The final selection of specialized care organizations per network together looked after between 82% and 98% of all juveniles residing in that municipality who received specialized care in the years 2017 or 2018. In this way, we were able to combine a representative participation of the specialized youth care organizations with a questionnaire that was manageable for all respondents [36, p. 82, 37, p. 4, 62, p. 31]. The networks included organizations from various sectors performing different tasks. Organizations that exchange (early warning) signals of support needs by children, youth and families with other organizations in the network have a signaling task. Gatekeepers are organizations that are legally authorized to refer clients to child and youth services covered by the Child and Youth Act [36, p. 84, 37, p. 7]. Organizations tasked with providing services deliver various child and youth support and care services. Table 1 presents the different sectors, the division of tasks and gives examples of organizations and professional groups that belong to a sector [36, p. 85, 37, p.3, 62, p. 31].

**Table 1:** Sectors, task division and examples of organizations in the network

Sectors	Tasks	Examples of organizations
1. Center for youth and family	gatekeeper	child and youth welfare and healthcare center
2. Municipality	signaling	youth care expert team, youth and family team*, school attendance officers, youth/social support/community service/employment/safety/purchase & contracting departments of the municipality
3. Basic social organization	signaling providing services	social work, welfare work, support for the disabled, youth and family support, library, food bank, refugee council
4. Education	signaling	care coordinators primary and secondary education
5. General practitioners	gatekeeper	child and family doctors
6. Health and prevention**	signaling gatekeeper	child and youth health care center, infant welfare center
7. Childcare and nursery	signaling providing services	pre-school, child day-care center, nursery, after school-care including homework support
8. Specialized youth care	providing services	youth mental health care, child and youth care, (forensic) psychiatry, orthopedagogy, psychology, care for disabled children
9. Protection & social rehabilitation	providing services	youth protection, youth probation officers, juvenile social rehabilitation
10. Safety	signaling providing services	police officers responsible for juveniles, prevention of child maltreatment, safety houses (crime prevention), public prosecution service, family & youth court, juvenile detention, childcare & protection board, community service supervisor
11. Volunteer organization	signaling providing services	Village or ward council, social policy advisory council, informal help for family or neighbors, community center, scouting/music/sport/leisure clubs

Note(s): * Youth and family teams also provide support services. ** The gatekeeper organization child health care is part of the sector health and prevention.

The three networks showed the same composition of organizations in terms of sectors. Network I, with 135 and 132 participating organizations in 2018 and 2019, respectively, is the largest network compared to Network II with 86 and 67, and Network III with 75 and 73 organizations, respectively. All sectors as listed in Table 1 are present in the networks, except for volunteer organizations in Network II since the network manager did not list them as network members [37, p. 3, 62, p. 31]. In 2018, the number of responding network members of Network I, II and III was 70 (52%), 49 (57%) and 51 (68%), respectively. In 2019, the response rates of Network I, II and III were 77 (58%), 39 (58%) and 44 (60%) organizations, respectively [37, p. 4, 62, p. 31]. Apart from the general practitioners, all the expected core network members responded. Most of the non-responders were network members that were expected to be at the network periphery, such as the municipality’s department of safety, organizations for childcare and nursery, or organizations for youth protection & social rehabilitation [36, p. 90, 37, p. 8, 62, p. 31].

### Measurement

The strength of the relationship was measured with a combination of the dimensions of frequency, reciprocity and multiplexity [23–25]. To measure the frequency of the contact between the organizations, the respondents were presented a list of all the organizations of the network and were asked to identify the organizations with which their organization had contact. Then, they were asked to indicate the frequency of this contact, on a four-point scale: several times a year - several times a month - several times a week - (almost) every day. Subsequently, to measure the reciprocity in the contact, the type of resource exchange was measured. That is because, for example, clients can be referred to another organization by just a care assessment decision without the active participation of the other organization, while both organizations need to actively participate in the interaction to exchange knowledge-based information with each other. The respondents were asked to indicate whether their organization had contact with the other organizations specifically for sharing expertise and knowledge (verbal case reports, and interprofessional consultation regarding clients’ conditions and effective treatment) and/or regarding client referral. The strength of a relationship is also determined by multiplex relations. Organizations that exchange multiple resources with each other are connected in more than one way [25]. When one resource exchange relation stops, they are still connected to each other. Therefore, multiplex relations are stronger than relationships that exchange a single resource. Table 2 shows which combination of dimensions was used to indicate a strong relationship.

**Table 2:** Indication of strong relations based on frequency, type of resource exchange (reciprocity) and multiplexity

Type of resource exchange (reciprocity) and multiplexity	Frequency			
	Daily	Weekly	Monthly	Annual
Expertise & knowledge sharing and client referral	**strong**	**strong**	**strong**	
Expertise & knowledge sharing	**strong**	**strong**		
Client referral				

Table 2 shows, for example, that a relation is considered strong if two organizations share their expertise and knowledge weekly. In addition, a relation is considered strong if two organizations engage in both expertise and knowledge sharing and client referral on a monthly basis.

To detect the critical relations in the overall structure of the networks we used the Lambda set approach. In this approach, each of the relationships in the network is ranked in terms of importance by evaluating how much of the resources flow among organizations in the network pass through each link. The relationship between two organizations which, if disconnected, would most significantly disrupt the flow among all of the actors is referred to as the Lambda set, or the most critical relation [29].

To measure the stability of strong relations - i.e., whether the strong relations between the individual organizations in 2019 were the same as those in 2018 – we used the QAP (quadratic assignment procedure) correlation procedure to calculate the overlap between the strong relation network structures of the networks in both years. QAP identifies the extent of the association in situations where there is no systematic connection between the two networks [29]. It compares the observed matching rate of the same type of relationship across two data collection periods (having the same nodes) to the average of a large number of trials in which the actors in the network are randomly matched [39]. As the relations are binary, we used the Jaccard Coefficient. Scores range between 0 and 1, with 0 indicating no overlap and 1 complete overlap between the networks [29].

### Data analysis

To analyze the data, we used Excel, Ucinet and Visone [69, 70]. The latter was mainly used to visualize the network graphs. First, we selected the relational data (frequency and resource exchange) of only the organizations that are members of the networks in both years (respectively 119, 65 and 71 organizations in Network I, II and III) and converted it into adjacency matrices in Excel. We used this selection for the analysis, as statistical tests to compare network structures over time requires networks with the same actors [29]. Moreover, to reflect relationships reported by each organizational dyad, and in that way to capture all links, the networks were symmetrized [69 pp. 352, 71]. This method examines unconfirmed or unidirectional network relations, which are relations where a respondent identifies a link between their own and another organization, but the other organization does not confirm this collaboration (including non-response) [70 pp. 350-351, 72]. We applied the following rule to create the adjacency matrices: a relation between two network members was coded as existing if at least one of the (boundary spanners of the) network members indicated this relation. The missing values were entered as a reciprocal relationship per responding organization (i.e., transposing the column in an adjacency matrix with the corresponding missing rows). This method is known as the procedure of labeled reconstruction [73] to manage non-response.

Subsequently, the adjacency matrices (frequency and resource exchange) were added together and the relations that were identified as strong were selected (see Table 2 for selection criteria). After inserting these adjacency matrices of strong relations in Ucinet, we computed the multiple network measures (number of active organizations, isolates, relations, average degree centrality, i.e., average number of strong relations per organization in the network, and Lambda sets) per network. Then we inserted the adjacency matrices in Visone to visualize the graphs of the networks regarding strong relations. In the graph, we used various shapes for the nodes to show the different sectors, a bigger size for the nodes of the gatekeeper organizations and a thick line for the relations that are Lambda sets.

Finally, to examine the stability of the strong relations - i.e., to what extent the strong relations between the individual organizations in 2019 were the same as those in 2018 – we ran the QAP (quadratic assignment procedure) correlation procedure of Ucinet for the whole networks and separately for the gatekeepers. After that, to visualize the graphs of the networks regarding stable strong relations, we merged the adjacency matrices of 2018 and 2019 into adjacency matrices of stable strong relation and inserted those in Visone.

## Results

The number of organizations that were members of the networks in both years was 119, 65 and 71 in Network I, II and III, respectively. All the sectors, including the gatekeeper organizations - as presented in Table 1 - were present in this selection. Most of the organizations that did not occur in both years belong to the specialized youth care sector (92%).

### Strong relations structure and critical relations

Table 3 presents the descriptive statistics for network structures regarding strong relations for organizations that are member of the networks in both years.

**Table 3:** Descriptive statistics for the network structures regarding strong relations in 2018 and 2019

	**Network I**		Network II		Network III	
	2018	2019	2018	2019	2018	2019
Number of organizations	119	119	65	65	71	71
Number of organizations with strong relations (% of all organizations)	100 (84%)	100 (84%)	52 (80%)	55 (85%)	57 (80%)	58 (82%)
Average degree centrality (range)	7 (0–54)	8 (0–61)	5 (0–28)	6 (0–31)	9 (0–38)	9 (0–41)
Number of strong relations (% of all relations in the network)	782 (30%)	928 (32%)	304 (29%)	392 (37%)	604 (41%)	614 (41%)
*Number of strong relations per gatekeeper organization*:						
Center for youth and family	56	61	15	31	30	30
General practitioners	16	22	13	11	19	13
Child health care	38	35	6	14	37	32

**Fig. 1:**
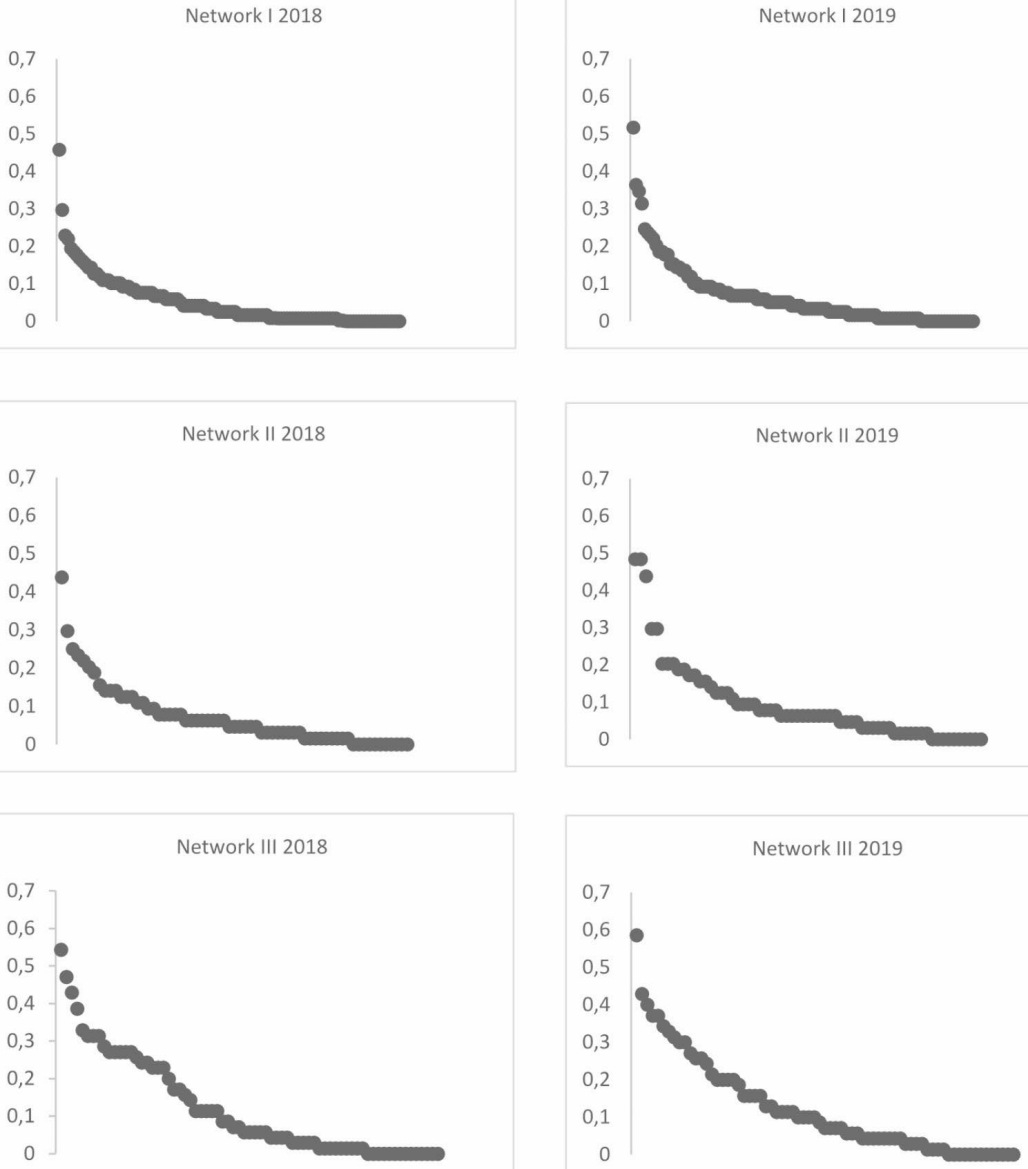
Scatter plots based on organizations’ degree centrality of strong relations per network in 2018 and 2019

As can be seen in Table 3, in both years a large majority of the organizations in the networks had strong relations (80–85%). Organizations without strong relations are mainly specialized youth care organizations and a few organizations from the municipality, childcare and nursery and basic social organization sectors (not in Table 3). Organizations have strong relations with an average of five to nine other organizations (range 0–61). In 2018 and 2019, respectively, the proportion of strong relations in Network III (41%, 41%) was the largest compared to Network I (30%, 32%) and Network II (29%, 37%). In both years, in all three networks, the organizations with a gatekeeper function had strong relations. In Network I and II, the center for youth and family has the strongest relations of the gatekeepers in the network, while in Network III this is child health care. Compared to the average number of strong relations per organization (five-nine organizations), most of the gatekeepers had many strong relations with other organizations. In particular, the center for youth and family in Network I had many strong relations. In 2018 and 2019, it had 56 and 61 strong relations with other organizations, respectively. This means that a small number of organizations is responsible for a majority of the strong relations in the network while the majority of organizations has just a couple of strong relations. Figure 1 shows these power law distributions of strong relations in all three networks at both measurement points. In the scatterplots, the organizations in the networks are on the X-axis and their degree centrality score is on the Y-axis.

**Fig. 2:**
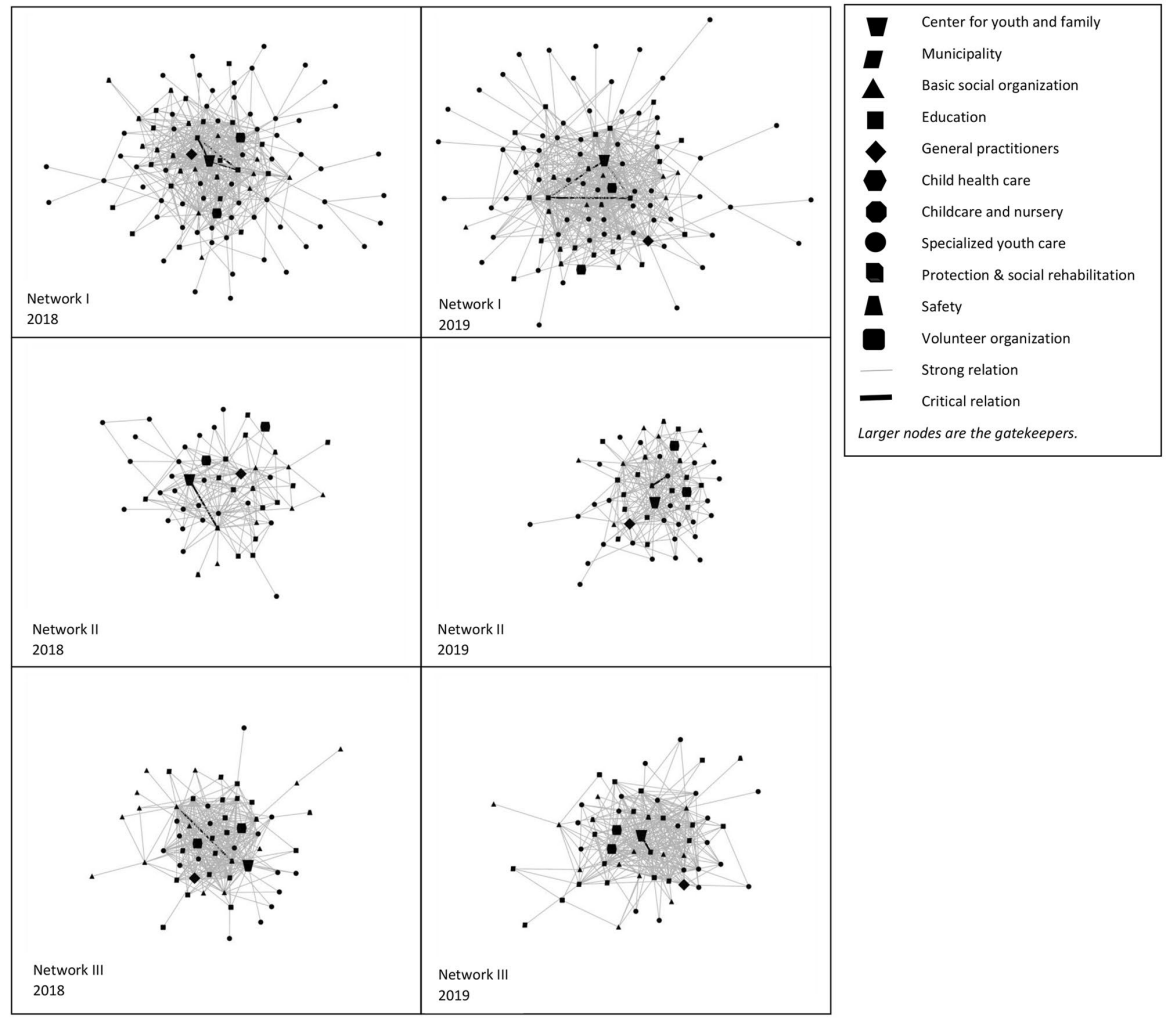
Structure of strong relations networks and critical relations in 2018 and 2019

Figure 2 presents the network diagrams of the strong relations networks in 2018 and 2019. The different shapes of the nodes show the sectors to which the organizations belong. The nodes (organizations) and lines (relations) that are bigger in size are respectively the gatekeeper organizations and the critical relations in the network structure.

Comparing the three networks in Fig. 2 clearly shows that Network II has fewer strong relations than Networks I and III. In all networks, most of the gatekeepers have a more central position in the network. Compared to the other gatekeepers, the child health care organizations in Network I in 2019 and in Network II in 2018 have a more peripheral position. Moreover, of the gatekeepers, the center for youth and family is the only one that has a key position, as it often forms a critical relation. The organizations with which the centers for youth and family have critical relations are school attendance officers, organizations for the prevention of child maltreatment, youth and family teams, care coordinators for secondary education, and organizations for youth protection & social rehabilitation. In Network II in 2019 and Network III in 2018, the critical relations are between organizations that are not gatekeepers. In both cases, it is the organization for the prevention of child maltreatment that held the key position in combination with youth and family support, and disabled childcare.

### Relation stability

More than half of the organizations, including the gatekeepers, had strong relations that were stable across time (59-66%). Across all the sectors as presented in Table 1, the number of organizations with stable strong relations was 73, 43 and 42 in Network I, II and III, respectively. The internal dynamics were examined by calculating the overlap between strong relation structures in both years and in particular the dynamics of the strong relations of the gatekeepers. Table 4 presents the results of the QAP correlation procedures.

**Table 4:** QAP Jaccard correlation between strong relationships regarding expertise & knowledge sharing and client referral in 2018 and 2019 for (gatekeeper) organizations that are members of the networks in both years

	Network I (N119)	Network II (N65)	Network III (N71)
All organizations	0.290**	0.386**	0.390**
Center for youth and family	0.463**	0.394**	0.667**
General practitioners	0.188**	0.333**	0.391**
Child health care	0.521**	0.333**	0.500**

**p < .01 (two-tailed, 2500 permutations)

There are statistically significant correlations between the strong relation structures over time and between the strong relation structures of the gatekeepers over time. In Network I, 29% of the strong relations between organizations in 2019 were the same as those in 2018. This means that over 70% of the strong relations in Network I were lost in one year. For both Network II and Network III, 40% of the strong relations are stable over time. In Network I and III, the centers for youth and family and the child health care organizations had more stable strong relations than the general practitioners. This applies in particular for the center for youth and family in Network III, with 67% of its strong relations remaining stable across time.

**Fig. 3:**
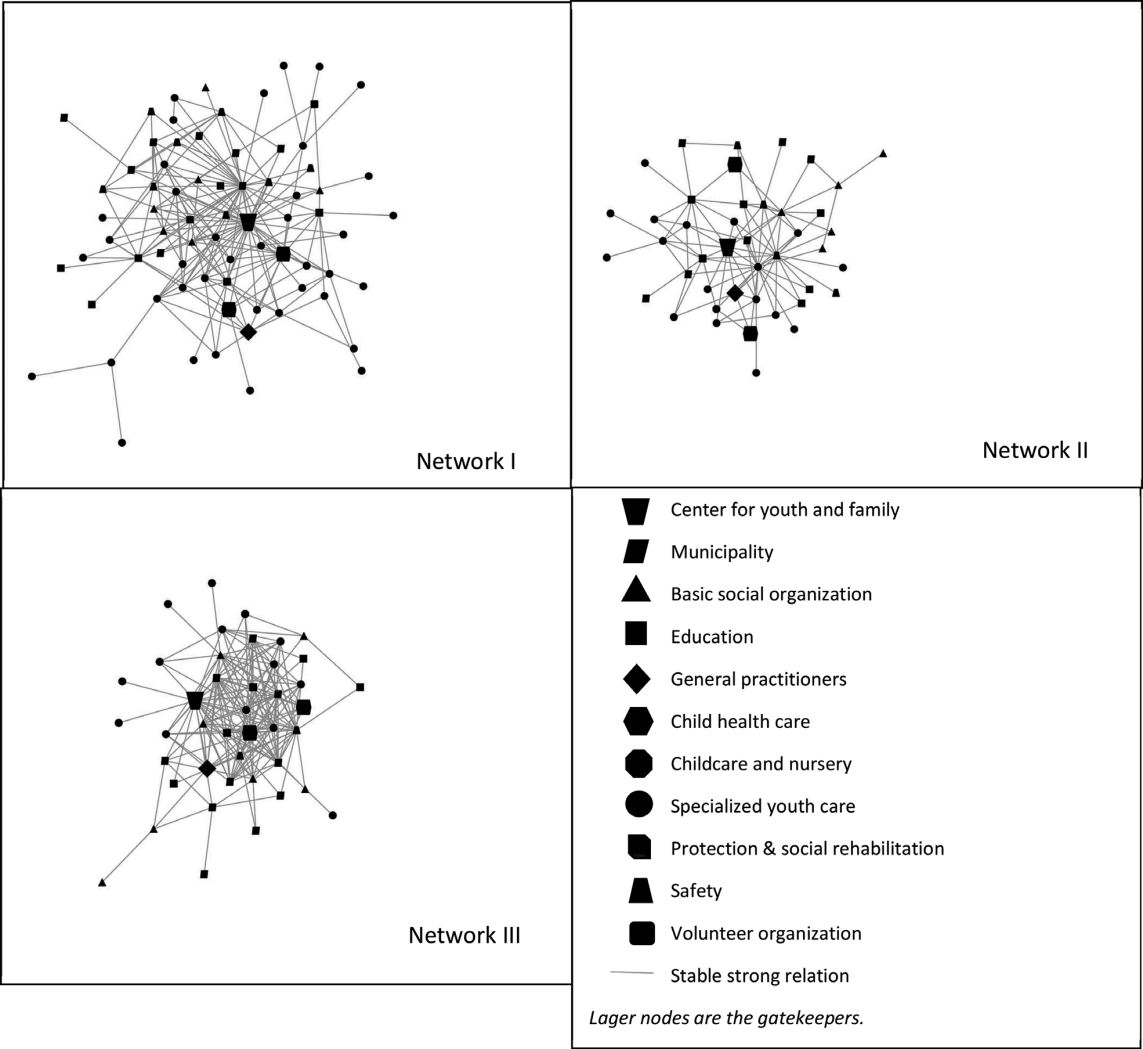
The stability of strong relations between 2018–2019

Figure 3 presents the network diagrams of the stable strong relations across time. The total number of stable strong relations in Network I, II and III were 384, 194, and 342, respectively. As the number of nodes reveals, the total number of organizations with stable strong relations in Network II and III are comparable. However, the number of lines in the diagrams shows that, between 2018 and 2019, Network III had more stable strong relations than Network II.

## Discussion

This study examined the strong relations structure, the critical relations and internal dynamics of three child service networks. Particularly, we assessed the strong relations of the gatekeeper organizations, i.e., the centers for youth and family, general practitioners and child health care organizations. Results show that more than 80% of the organizations within child service networks have strong relationships with other organizations. All gatekeepers are included in the strong relations structure. On average, an organization has strong relationships with 5–9 organizations. However, the strong relations are very unequally distributed across the organizations. In all three cases at both measurement points, a small number of organizations is responsible for the majority of the strong relations in the network. We found that most of the gatekeepers maintain an extremely high number of strong relationships within the network. The center for youth and family in Network I, for instance, had strong relations with 61 organizations in 2019. Due to this high number – combined with the center’s limited resources, energy and time – there is a serious risk of inefficient and ineffective functioning of the network as a whole [29]. In all three cases, the center for youth and family holds a critical relationship in the network at, at least, one measurement point. This means this center has a key position in the network: it controls the most important relations in the networks, and most of the resources flowing between organizations in the networks run through this critical relation [29].

The development over time shows that child service networks are highly dynamic systems. Despite more than half of the organizations having stable strong relations, the individual strong relationships within the networks appear to be rather unstable. With a loss of 60 to 70% of the strong relations in a year, strong relationships in the networks are clearly subject to major internal network dynamics. For example, the strong relations of the general practitioners with other organizations were relatively unstable; in 2019, only 19 to 39% of their strong relations with other organizations were the same as in 2018. Since unstable relationships jeopardize the exchange of more fine-grained and tacit information regarding clients’ conditions and effective treatment, the found instability is relevant [26–28]. A notable finding is the relatively high stability of the strong relations of some of the gatekeepers. Compared to all the strong relationships in the networks (30–40%), the strong relations of the centers for youth and family and the child health care organizations in two of the three networks are relatively stable over time (47–67%).

The time between the two measurement points was about one year, which might be rather short to examine developments over time properly. At the same time, the internal dynamics have become visible over the course of a single year. That is striking, since the research started three years after the decentralization of the key responsibilities for child welfare and healthcare service delivery from the central to local levels of government: a period previously indicated as sufficient time for networks to stabilize [74]. Apparently, strong relation structures need more than three years to regroup after such a major shakeup of the system. The found instability of strong relations within the networks is relevant, as the child welfare and healthcare state reform was precisely meant to strengthen the relations between the various child service organizations [11, 13, 14]. To examine whether the time required to stabilize is longer for strong relationships or whether these relationships are always flexible, further research should be longitudinal with several measuring points in time or at least a longer period than one year between the two measurements [37]. Also, the case study design should be used to also reflect upon the results of the quantitative network analyses.

Although strong and stable relations are crucial preconditions for integrated care, it is uncertain whether it is necessary to have strong relations between all the different organizations in the network or whether it is sufficient for the gatekeeper organizations and some of the organizations per sector (see Table 1) to have strong relations. In terms of network governance, the latter would imply a hub and spoke structure, whereby one central gatekeeper organization is connected to a smaller core group of organizations which function as brokers to the peripheral organizations in the network [75]. This especially applies to the centers for youth and family, as these centers were specifically formed – on account of the child welfare and healthcare state reform – to become the hub between preventive support (e.g. basic social organizations, education, health and prevention, childcare and nursery, volunteer organizations), primary care (e.g. child healthcare, general practitioners, social work, youth and family support) and specialized care (e.g. specialized youth care, protection and social rehabilitation, safety organizations) [12, 14, 36].

The found combination of considerable instability of strong relations at the whole network level and the fairly high stability of strong relations of (part of) the gatekeepers (at the organization level) highlights the contradictory logic of desired stability and flexibility [75]. On the one hand, networks strive for relationship stability, as this is critical to maintaining legitimacy inside and outside the network. Moreover, the stability of relationships of core organizations appears to be a major factor in explaining network effectiveness regarding client services, especially in case of vulnerable client populations [76]. On the other hand, relationship flexibility on account of new task demands gives networks their advantage over vertically integrated organizations, which can be rigid and bureaucratic [60, 75].

Thus, the considerable instability of strong relations can also be seen as the flexible operation of strong relations networks. This flexibility is essential for the delivery of comprehensive, tailor-made services. Indeed, instead of routinely referring clients, gatekeepers need to refer clients in a targeted manner so that children and families in need get the most appropriate support and services, and that requires a higher relationship turnover of strong relations. At the same time, our study shows that the gatekeepers – or at least the most important gatekeeper, i.e., the centers for youth and family – have stable strong relationships that connect the large diversity of service organizations to form an interconnected network, i.e., a hub and spoke structure. This stability is essential to successfully perform core functions such as early-warning signaling, triage, service delivery, client referral, and interprofessional consultation [3, 4, 19, 25, 42, 43].

However, this setup would still mean that the centers for youth and family need to maintain a fairly large number of strong relationships with a core group of broker organizations that at least represent the ten other sectors in the youth care system. Since the decentralization was accompanied with an overall cost reduction, it could be quite possible that these typically larger organizations do not have resources specifically dedicated to build and maintain strong ties. Network managers should realize that even a more centrally organized child service network demands extra attention, time and resources to achieve the integration necessary to successfully accomplish a cohesive youth care system that facilitates integrated care in families’ own environment [59]. Further research should examine what the maximum number of strong relations is that such an organization and a network as a whole can efficiently and effectively build and maintain. Specifically, it should address what additional effort – attention, time and resources – is required to build and maintain a successfully functioning strong relations network [59].

For this study, some methodological remarks can be made. First, our focus on Dutch child service networks may limit the generalizability of our findings. However, since we used a broad context and many other countries have also implemented governance reforms including a decentralization of health systems, our results are probably also applicable to other contexts and countries [5, 7, 8, 10]. Second, to obtain the maximum amount of information, we used the whole network approach. After all, whole network data allows for very powerful descriptions and analyses of social structures [29]. In order to reflect relationships reported by each organizational dyad and to apprehend any link, the networks were symmetrized [71]. However, this means that we examined unconfirmed ties, which may have led to an overestimation of some network relations. Specifically, the relations of the non-response organizations need to be interpreted with caution. Fortunately, the responders included all the expected core network members, with the exception of the general practitioners. That is positive, as the greatest bias in most network measures occur if more central organizations are missing, and the least bias if peripheral organizations are missing [77]. Most of the non-responders were network members at the periphery of the network.

## Conclusion

By examining the structure and dynamics of strong interorganizational relationships from a network perspective, this study addressed crucial preconditions for integrated care. The child service networks have appropriate strong relations structures. The important gatekeepers have key positions and their strong relations are relatively stable. Around these core organizations, there is a large diversity of service organizations with flexible strong relations. However, the extremely high number of strong relations that particularly gatekeepers need to build and maintain, in combination with the considerable instability of strong relations considering the whole network, is a serious point of concern that needs to be addressed by the management of the network.

## Data Availability

The data underlying this article cannot be shared publicly to protect the privacy of the participants but are available from the corresponding author on reasonable request.

## Declarations

### Authors’ information

M.B. is PhD student/science practitioner. Her research focuses on the functioning of large cross-sectoral organizational networks for child welfare and healthcare service delivery. J.M. is Professor in Care for Youth at Tilburg University. Her research focus is on child, parent and counsellor factors, which influence the results of care. CH.N. is clinical psychologist/psychotherapist and Professor in Forensic Mental Health Care, and Professor in Transforming Care for Youth at Tilburg University. J.R. is Professor in Policy and Organization Studies at Tilburg University. His research focuses on the governance and effectiveness of organizational networks. H.O. is Professor in Public Health at Tilburg University. His research focus is on public health epidemiology and bridging the gap between science and policy in public health.
